# Application of the LPE-Grown LuAG: Ce Film/YAG Crystal Composite Thermoluminescence Detector for Distinguishing the Components of the Mixed Radiation Field

**DOI:** 10.3390/ma15248708

**Published:** 2022-12-07

**Authors:** Anna Mrozik, Paweł Bilski, Wojciech Gieszczyk, Mariusz Kłosowski, Sandra Witkiewicz-Łukaszek, Vitaliy Gorbenko, Tetiana Zorenko, Yuriy Zorenko

**Affiliations:** 1 Institute of Nuclear Physics Polish Academy of Sciences, 31-342 Krakow, Poland; 2 Institute of Physics, Kazimierz Wielki University, 85-064 Bydgoszcz, Poland

**Keywords:** thermoluminescence, composite detectors, garnets, liquid-phase epitaxy, crystals, single-crystalline films

## Abstract

Single-crystalline films (SCFs) of the LuAG: Ce garnet grown using the liquid-phase epitaxy method onto YAG single-crystal (SC) substrates were investigated for possible applications as composite thermoluminescent (TL) detectors. Such detectors may help to register the different components of ionizing radiation fields with various penetration depths, e.g., heavy charged particles and gamma or beta rays. It was found that the TL signal of LuAG: Ce SCF sufficiently differs from that of the YAG substrate concerning both the temperature and wavelength of emissions. Furthermore, even by analyzing TL glow curves, it was possible to distinguish the difference between weakly and deeply penetrating types of radiation. Within a test involving the exposure of detectors with the mixed alpha/beta radiations, the doses of both components were determined with an accuracy of a few percent.

## 1. Introduction

The composite detectors have been well known for many years in the form of phoswich scintillators (phosphor sandwich) for the active-mode (in situ) registration of ionization radiation [1,2,3,4,5]. The main application areas of such detectors are environmental radiation monitoring for the registration of different components of mixed ionizing fields, nuclear physics for an analysis of the particles with different masses, and pathways in detector materials as well as in biological and medical microtomography as multi-layered X-ray image screens with a high spatial resolution [6,7,8,9,10,11,12,13]. The composite detectors could also be applied in nuclear medicine, where the emitters of alpha and beta particles are used. It could be recognized also in oncology for the testing of proton and X-ray beams with different energies. Finally, such composite detectors could be used to diagnose alpha/gamma radioactive sources which can produce mixed radiation fields.

To separate the signals coming from both parts of such composite detectors, the main differences in scintillating decay kinetics are used [14,15]. However, the application of the active-mode registration of ionization fluxes in situ using scintillators is not always possible, especially in the case of extremely low or high doses of radiation and long-time radiation exposure. Such problems demand another approach to be considered in order to produce composite detectors of ionization radiation. Namely, we also consider the simultaneous registration of various components of mixed ionization fluxes using differences between the TL glow curves, coming from the film and substrate parts of a composite TL detector (passive mode of registration).

Recently, we developed the first prototypes of composite TL [16] detectors based on the epitaxial structures of garnet compounds produced using the liquid-phase epitaxy (LPE) method [17]. Namely, in the previous work, we introduced LPE-grown YAG:Ce SCF/YAG crystal and LuAG:Ce SCF/YAG crystal epitaxial structures and analyzed their TL properties after irradiation via α and β particles [17]. In this work, we introduce the results of the extended research for LuAG:Ce SCF/YAG crystal epitaxial structures which also possess the ability to distinguish the difference between SCF and crystal signals at the registration of various types of ionization radiation, namely particles and X-rays quanta.

The advantages of such types of composite TL detectors based on the different types of garnets and other oxide materials in comparison with conventional analogues (LiF, Al_2_O_3_: Ce [18,19,20,21]) were connected with their universality for the registration of various types of ionization radiation, together with their excellent radiation stability, the uniformity of structural and optical properties, the high yield of TSL, and the good position of the main glow peaks around 290–300 °C. The application of the LPE method also opens up a rich perspective for the creation of “all solid-state composite TL detectors” based on materials with different densities.

The samples of the composite detectors with different thickness layers were tested. This research is new and the proposed approach to discuss results seems to show a new path of the detection system and interpretations of the signals. Additionally, an attempt to quantitatively determine TL signals which originate from different components of a mixed radiation field was made.

## 2. Materials and Methods

### 2.1. Materials under Study

**Figure 1:**
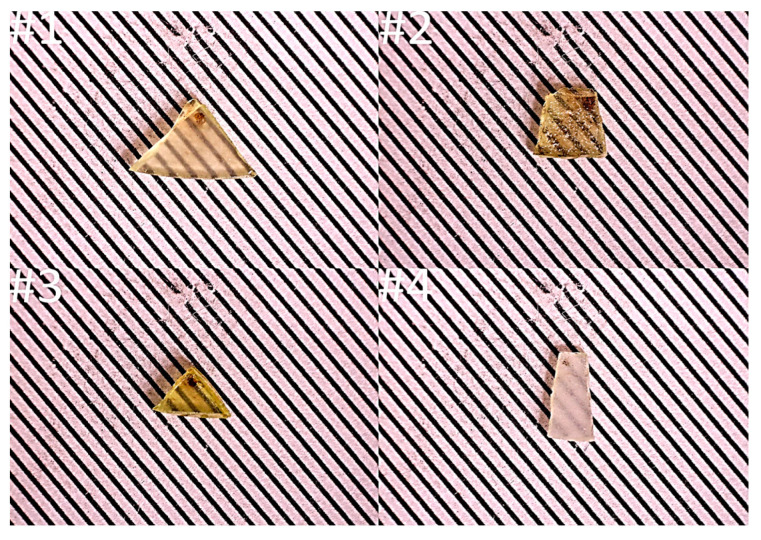
The samples under study: #1 LuAG:Ce SCF/YAG SC, h_SCF_ = 15 µm; #2 LuAG:Ce SCF/YAG SC, h_SCF_ = 37 µm; #3 LuAG:Ce SCF/YAG SC, h = 78 µm; #4 YAG SC substrate (undoped).

The materials under study consisted of composite detectors based on the LPE-grown epitaxial structures of garnets. The LuAG: Ce SCF/YAG SC epitaxial structures consisting LuAG: Ce films with thicknesses in the 15–78 μm range were grown from the melt–solution based on the PbO–B_2_O_3_ flux onto the nominally undoped YAG substrates, prepared from the crystals grown by the horizontal direct crystallization (HDC) method (see Figure 1 and Table 1).

The structural properties of the epitaxial structure are a very important characteristics of the quality of detectors. In several previous papers [16,22], the detailed results of XRD and TEM investigations of LuAG: Ce/YAG epitaxial structures, including the determination of the SCF/substrate misfit, the investigation of the SCF/substrate interface, etc., have been presented. The main topic of the presented paper is related to the TL properties of the chosen type of epitaxial structure and the other results of research on epitaxial structures with close composition published in [16,23].

### 2.2. Irradiation Sources

Investigations of the properties of composite detectors required the application of both weakly and strongly penetrating radiation. The first one included alpha particles from the ^241^Am source and soft X-rays, while the latter consisted of ^90^Sr/^90^Y beta particles (1.4 GBq source).

The spectral measurements required very high doses due to the lower sensitivity of the spectrometer compared to the PMT tube or the CCD camera. The alpha particle irradiations had to last several days. The ^241^Am source had a low activity (10.7 MBq); thus, during the experiments, the alpha particle irradiations had to last for several days. The nominal energy of ^241^Am alpha particles was 5.486 MeV, but in relatively thick sources of this type, the energy degraded to a broad spectrum (1 MeV to 4.5 MeV) with a peak at 3.5 MeV [24]. Besides alpha particles, an ^241^Am source also emits 60 keV gamma rays.

An additional source of weakly penetrating radiation, which was applied during investigations, was a mini X-ray generator (needle-like anode X-ray tube) with a gold target and a Be window, produced by the National Centre for Nuclear Research in Warsaw [25]. It operated at a 15 kV voltage, which means that the maximum photon energy was 15 keV. Usually, the average energy of X-rays is assumed to be between ⅓ to ½ of the maximum energy, but in the spectrum of this X-ray tube, several characteristic L lines of Au were present between 9.63 keV and 13.38 keV [26], thus shifting the average energy. For simplification, we assumed the average energy to be 10 keV.

Some data relevant to the interaction of the used radiation types with the investigated materials are given in Table 1. The range of alpha particles with an energy of 3.5 MeV was calculated with the SRIM program [27]. The value of photon absorption for each sample was calculated by the formula:

(1) $$\frac{I}{I_{0}}=exp\left(-\left(\frac{\mu }{\rho }\right)\times d\right)$$

where *μ/ρ* is mass attenuation coefficient and d is the mass thickness (*d* = *x* × *ρ*, where *x*—thickness and *ρ*—density).

Values of the mass attenuation coefficient were obtained according to simple additivity:

(2) $$\frac{\mu }{\rho }=\sum w_{i}\times {\left(\frac{\mu }{\rho }\right)}_{i}$$

The value of *w_i_* is the fraction by weight of the *i*th atomic constituent. All of the values of ${\left(\frac{\mu }{\rho }\right)}_{i}$ were taken from the NIST database (https://physics.nist.gov/PhysRefData/XrayMassCoef/tab3.html, accessed on 9 May 2022).

### 2.3. TL Glow-Curve Measurements and TL Emission Spectra Measurements

**Figure 2:**
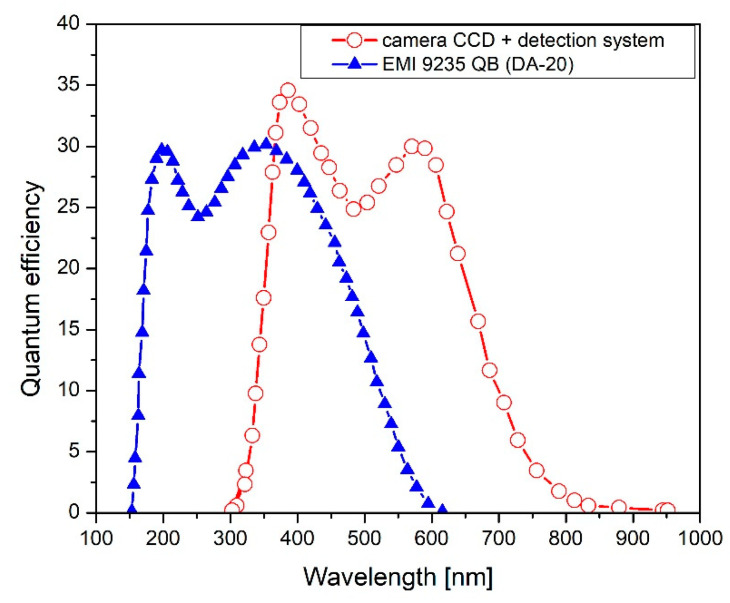
Comparison of spectral characteristics of the applied detection system: camera CCD (with lenses)—red circle, and photomultiplier EMI 9235 QB used in the DA-20 TL reader—blue triangle.

In this paper, the thermoluminescence method was applied. TL is a luminescence phenomenon that can be observed when the material is thermally stimulated after previous irradiation. TL can be explained on the basis of the band theory of solids. Radiation causes the ionization of valence electrons and the creation of electron–hole pairs. The presence of structural defects and impurities leads to the production of new local energy levels in the forbidden band. Heating of such an irradiated material leads to the absorption of energy by the trapped electrons and transitions from the trapping centres into the conduction band. A recombination of the released electrons with localized holes results in radiative emission and luminescence [28,29,30]. From the point of view of the application of TL detectors in dosimetry, these traps are important, which are energetically deep enough to be stable, i.e., to localize charge carriers for a sufficiently long time (‘storage time’). The spectrally resolved TL measurements were carried out with the automated Risø DA-20 TL/OSL reader. Normally, the detection system consists of a bialkali photomultiplier tube and a set of bandpass filters chosen appropriately to cover the emission range of the studied samples. In the current work, in place of the PMT, the Ocean Optics QE Pro 00689 spectrometer was mounted. The spectrometer allows TL emission spectra to be registered over the wavelength range from 200 to 1000 nm with a 4 nm resolution. While it is possible to also extract the standard TL glow curves (i.e., the relationship between the TL signal and the temperature) from the spectrometric data, the measurement sensitivity of the spectrometer is much lower than that of PMT, and therefore irradiations with very high-doses are needed. For this reason, as well as to be consistent with the conditions of practical dosimetry measurements, the TL glow curves were preferably registered with the dedicated TL readers. However, the emission spectra of the studied materials extend to the long-wavelengths, therefore making it impossible to use the standard Risø DA-20, which photomultiplier is not sensitive beyond c.a. 500 nm. Instead, the TL glow-curve measurements were carried out using the two-dimensional TL reader constructed at IFJ PAN [31]. The reader used the PCO SensiCamTM VGA CCD 12-bit camera, with a spectral range of 300 to 800 nm, equipped with a 60 mm diameter steel heater, for light detection purposes. Figure 2 shows a comparison of spectral characteristics of both readers. The temperature can be raised linearly up to the temperature of 400 °C, with heating rates ranging from 1 °C/s to 10 °C/s. The light emitted from the samples was registered over the entire temperature range up to 350 °C with a heating rate of 2 °C/s. It should be noted, however, that the sensitivity of the camera was much lower than that of standard PMTs.

## 3. Results

### 3.1. YAG Substrate

Before analyzing the TL of the composite detectors, the YAG SC substrates, used for the growth of epitaxial layers, were investigated. TL glow curves after ^90^Sr/^90^Y irradiation were registered, making it possible to identify which TL peaks of the composite detectors originate from the substrate.

**Figure 3:**
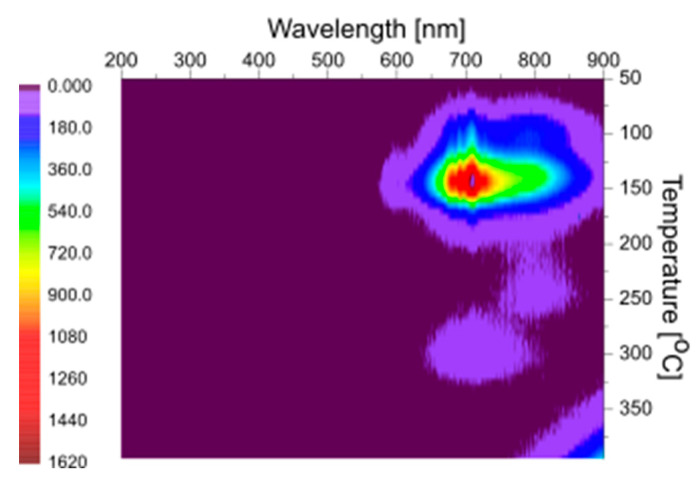
Emission spectra of the YAG substrate after beta-particle irradiation.

In Figure 3, the 2-D emission spectra of the YAG SC substrate, measured after irradiation with ^90^Sr/^90^Y source, are presented. The substrate sample shows TL emissions in the range of 500 nm up to 900 nm. The origin of this emission is not clear nowadays, but it is most probable that this emission center is formed by the complex defect of YAG crystals, namely by the aggregate of the charged oxygen vacancies [32,33,34,35,36,37,38,39,40].

**Figure 4:**
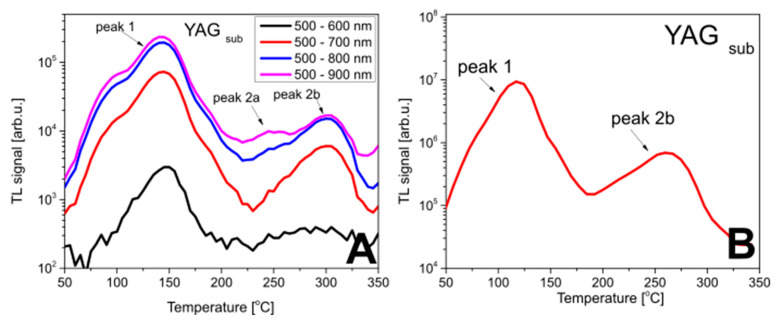
TL glow curves of the YAG substrate after irradiation with ^90^Sr/^90^Y measured with the Ocean Optics QE Pro 00689 spectrometer (**panel A**) and with the TL reader utilizing a CCD camera (**panel B**).

The temperature data indicate that three peaks at different temperatures can be observed. The number of visible peaks, however, strongly depends on the wavelength integration range (Figure 4, panel A). Only for the 500–900 nm wavelength range can all three peaks be seen. Regardless of the width of the spectrum that was considered, the substrate gives signals both in the low- and high-temperature areas, and the signal above 200 °C has a lower intensity than the low-temperature area peaks.

In this study, the TL reader with a CCD camera was used for TL measurements, capable of registering TL in the wavelength range of 300 to 700 nm. This means that only two main peaks are visible in the measured TL glow curve (Figure 4, panel B), as peak 2, whose emission extends beyond 800 nm, is missing.

### 3.2. ^241^Am Irradiation

**Figure 5:**
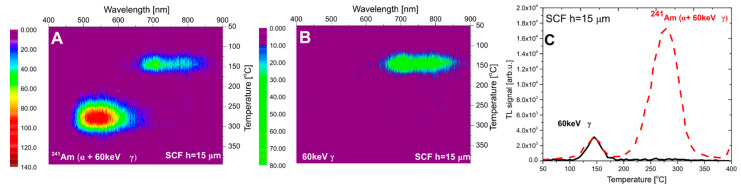
The emission spectra of LuAG: Ce SCF/YAGSC (h_SCF_ = 15 µm) (#1) after irradiation with ^241^Am (**panel A**—irradiation with the bare source (α + 60 keV γ), **panel B**—irradiation with the covered source (only 60 keV γ), **panel C**—TL glow curves taken from spectral measurements (integration in range 400–700 nm)).

**Figure 6:**
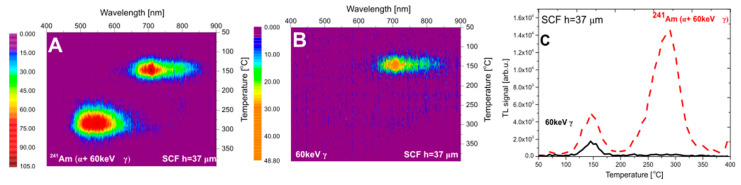
The emission spectra of LuAG: Ce SCF/YAG SC (h_SCF_ = 37 µm) (#2) after irradiation with ^241^Am (**panel A**—irradiation with bare source (α + 60 keV γ), **panel B**—irradiation with covered source (only 60 keV γ), **panel C**—TL glow curves taken from the spectral measurements (integration in range 400–700 nm)).

In the next step of the investigations, the TL emission spectra of the LuAG:Ce SCF/YAG SC composite detectors were studied, following irradiation with the ^241^Am source. The results for samples #1 and #2 are presented in Figure 5 and Figure 6, respectively. Sample #3 was found to exhibit too low TL signal after alpha exposure to be measured by the spectrometer (we were able to measure its spectra after X-ray irradiation—see Section 3.3). The alpha particles range in the LuAG host is 7.80 µm (see Table 1); thus, one can be certain that α particles can be fully stopped in the SCF part of the detector.

In both figures (panel A), an emission band in the 450–650 nm range is present. It is well known that the luminescence spectra of all Ce^3+^-doped garnets consist of a broad band in this range [41]. The cerium dopant is only present in the SCFs. These results confirm that this signal originates from the α-irradiated film part of composite detector. However, a second emission band in the range of 650–900 nm is also visible. This signal corresponds to the emission spectrum measured for the YAG crystal substrate after beta-particle irradiation (Figure 3). It is therefore reasonable to assume that this emission band originates from the substrate. While α particles cannot reach the substrate, one has to remember that also 60 keV photons are emitted by the ^241^Am source. In the case of materials with a low value of an effective atomic number (Z_eff_), the impact of this photon emission on a TL signal may be neglected. However, for materials with high Z_eff_, the situation is quite opposite and the influence of 60 keV gamma rays is evident [42]. To confirm that these photons are the cause of the observed long-wavelength emission from the composite detector, we repeated the irradiation with the ^241^Am source covered by 125 µm-thick Kapton foil, which ensured that all alpha particles were stopped, but had a negligible effect on gamma photons. All other exposure conditions (geometry, time, etc.) were kept constant. The measured TL emission spectra (panels B of Figure 5 and Figure 6) agree well with the previously observed 650–900 nm emission band, which confirms the impact of the dose deposited by ^241^Am gamma rays in the substrate YAG crystal. This is further illustrated by TL glow-curves presented in panel C of Figure 5 and Figure 6. It is clear that peak 2 (at 280–290 °C) is related to the thin layer (containing Ce dopants) and peak 1 (at 145 °C) originates from the substrate.

### 3.3. X-Rays and β-Particle Irradiation

In the next step of the investigation, the thermoluminescence of the composite detectors was studied following soft 15 kV X-rays and β particle irradiations. The values of the photon absorption for the estimated average energy of X-rays (10 keV) are given in Table 1.

**Figure 7:**
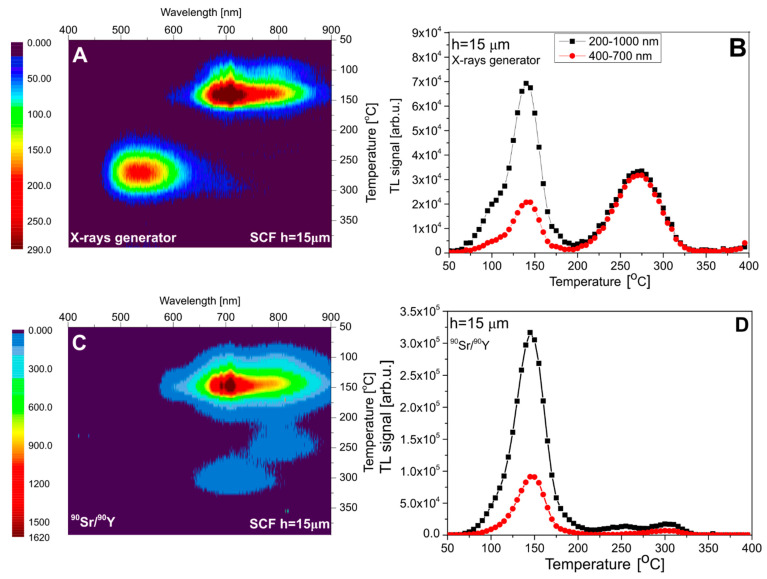
The emission spectra of sample #1 (**panel A**—after irradiation with 15 kV X-rays and **panel C**—after irradiation with ^90^Sr/^90^Y) and TL glow curves (**panels B**,**D**). TL glow curves are taken from the spectral measurements.

Figure 7 presents the results for sample #1, with an SCF thickness of 15 µm. In this case, roughly equal doses were deposited by X-rays in the SCF and in the substrate. The measured spectrum (panel A) agrees well with the expectations, showing two distinct emission bands at 700–800 nm (at low temperatures) and at 500–600 nm (at high temperatures), which correspond to the substrate and the SCF, respectively. Panel C of Figure 7 presents the spectrum measured after ^90^Sr/^90^Y beta-particle irradiation. This spectrum is nearly identical with that measured for the pure YAG crystal (Figure 3), with the absence of the Ce^3+^-related emission. This indicates that, in this case, the whole signal originates from the substrate and the dose deposited by beta particles in the thin SCF was too small to generate a measurable TL. Panels B and D of Figure 7 present TL glow curves obtained by the integration of spectral data within two ranges: 200–1000 nm (full range) and 400–700 nm, corresponding to the approximate spectral sensitivity of the TL reader with a CCD camera, used in further measurements. For clarity, the data of all discussed TL peaks are summarized in Table 2.

**Figure 8:**
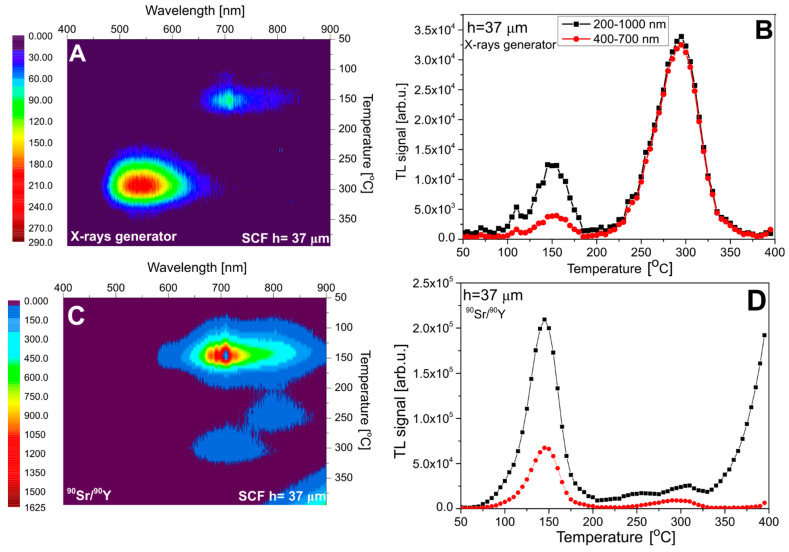
The emission spectra of sample #2 (**panel A**—after irradiation with 15 kV X-rays and **panel C**—after irradiation with ^90^Sr/^90^Y) and TL glow curves (**panels B**,**D**). TL glow curves are taken from the spectral measurements.

The analogous data for sample #2, with the SCF thickness of 37 µm, are presented in Figure 8. The one important difference with the previously discussed data concerns the emission band in the 700–800 nm range, which for X-ray exposure is for this sample very weak. This can be attributed to the obviously stronger absorption of low-energy photons in the SCF layer (see Table 1), which causes only a small dose fraction to be deposited in the substrate.

**Figure 9:**
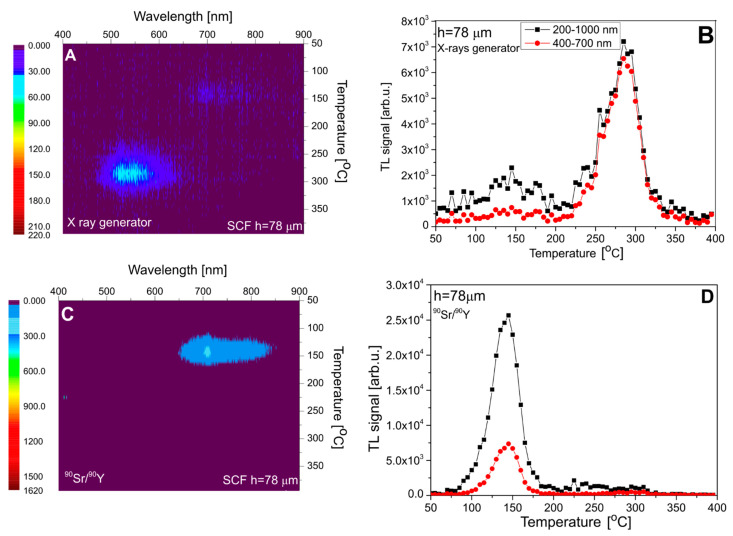
The emission spectra of sample #3 (**panel A**—after irradiation with 15 kV X-rays) and **panel C**—after irradiation with 90Sr/90Y) and TL glow curves (**panels B**,**D**). TL glow curves are taken from the spectral measurements.

Sample #3, as mentioned, exhibits much lower overall TL sensitivity; therefore, obtaining meaningful spectral data is difficult. Nevertheless, the spectra presented in Figure 9 are consistent with the general trend. As the thickness of the SCF in this sample is 78 µm, X-rays are fully absorbed in the SCF and cannot reach the substrate. Consequently, there is no emission band in the 700–800 nm region (panel A).

**Figure 10:**
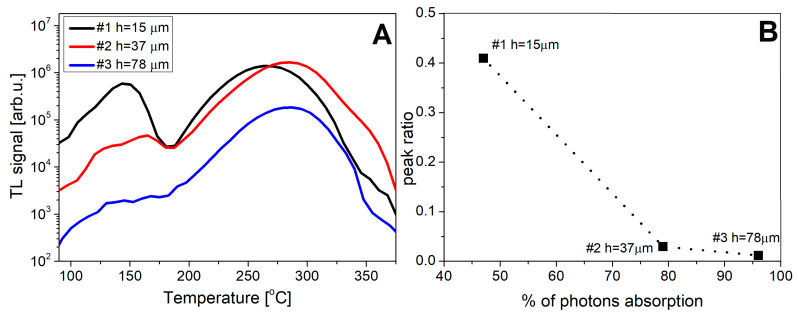
Analysis of TL glow-curve shapes of the investigated SCF samples after irradiation with X-rays (**Panel A**). **Panel B**—The relationship between the ratio of TL peak 1 to TL peak 2 and the percentage of the absorbed photons in SCFs after irradiation with 10 keV X-rays. TL glow curves were measured with the TL reader with a CCD camera.

Figure 10 presents the TL glow curves of the studied samples measured with the TL reader with a CCD camera. The results are consistent with the data obtained with the spectrometer. Panel D illustrates the dependence of the peak ratio on the thickness of the SCF layer, which in turn determines how many X-ray photons can reach the substrate. It can be also seen that the position of the maximum of peak 2 is located at different temperatures for different samples. The maxima of peak 2 are at 260, 282, and 289 °C in order to increase the SCF thickness (the peak positions somewhat differ from the values given in Table 2 due to a different heating system of the reader and the limited spectral sensitivity of the CCD camera). This shift is caused by a temperature lag during the heating of samples, which is bigger for thicker crystals.

### 3.4. TL Glow-Curve Deconvolution

Generally, the mechanism of thermoluminescence in the materials under study is connected with the liberation of electrons from the traps with different depths, following recombination on the emission centers formed by the dopant (Ce^3+^ ions in LuAG: Ce SCF) or host defect (YAG substrate).

In the simplified assumption, individual traps are assosciated with different peaks, which can be seen in the TL glow curve.

**Figure 11:**
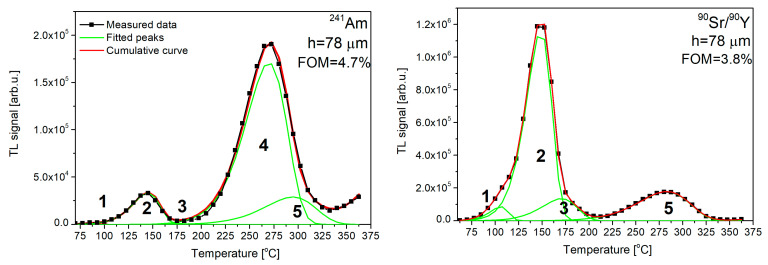
Exemplary results of the deconvolution performed for the studied LuAG:Ce SCF/YAG SC (h = 78 µm) after irradiation with different types of ionizing radiation.

The measured TL glow curves were deconvoluted into single first-order components using the Solver tool in Excel. The sample below the TL deconvolution (h = 78 µm) is presented (Figure 11). The parameters of different peaks are presented in Table 3 (for clarity of Figure 11).

The glow curves consist of several overlapping components. As can be seen, an acceptable fit, demonstrating a figure of merit lower than 4.7%, requires five peaks after irradiation with ^241^Am and five peaks after irradiation ^90^Sr/^90^Y. Based on the obtained fits, the peak parameters were estimated. Detailed information on calculated values, such as the peak position and energy trap depth, is shown in Table 3.

In the TL glow curve after irradiation with ^241^Am, peak No. 5 was related to 60 keV photons emitted by the ^241^Am source. The contribution to the TL signal from 60 keV photons was slight and was omitted in the calculations.

### 3.5. Mixed Radiation Field

The results discussed so far illustrate the differences between TL signals originating from the substrate and SCF, which are therefore related to deeply or weakly penetrating radiation, respectively. If TL measurements are spectrally resolved, then distinguishing between both components of the radiation field is easy, as they are visible as separate “islands” on the 2D temperature–wavelength plane. However, in practical TL measurements, only temperature-resolved data are normally available. This raises the question of whether distinguishing between radiation components based only on the analysis of the standard TL glow curves is possible.

To answer this question, a test of the real capability of the composite TL detectors to provide dose values of the components of an unknown mixed radiation field with acceptable accuracy was performed. In order to simulate such a quasi-mixed field, the samples were first irradiated with ^90^Sr/^90^Y beta particles (120 Gy) and then exposed to alpha particles (fluence 1.6 × 10^11^ cm^−2^). The TL measurements were taken with the reader equipped with the CCD camera.

**Figure 12:**
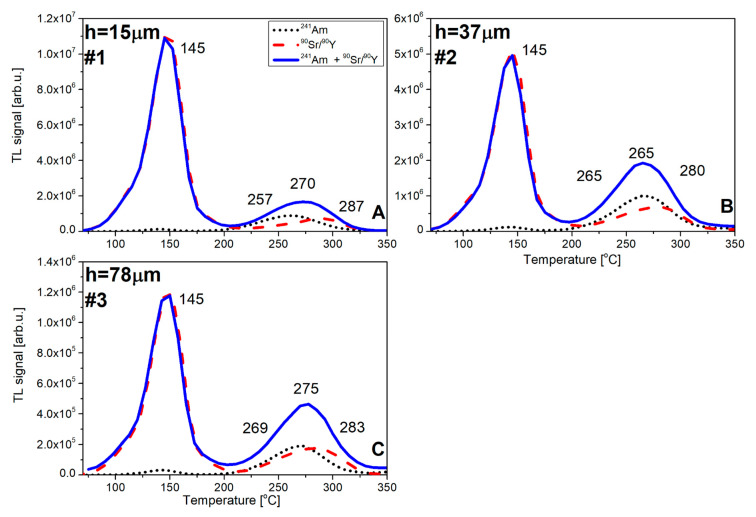
TL glow curves of LuAG: Ce SCF/YAG SC ((**A**)—h = 15 µm, (**B**)—h = 37 µm, (**C**)—h = 78 µm) after irradiation with different irradiation procedures (^241^Am, ^90^Sr/^90^Y, and mixed radiation). The values of fluence and dose are: Φ = 1.6 × 10^11^ [cm^−2^] and D_β_ = 120 [Gy], respectively.

Figure 12 shows the results of TL measurements after irradiation in such a quasi-mixed field. For clarity of interpretation, the glow curves following exposures to single radiation types (alpha or beta) are also presented. The glow curves had a typical shape with two apparent peaks. In agreement with the previous discussion, peak 1 was almost entirely due to beta-exposure, while peak 2 contained contributions from both radiation types (peak 2 and peak 2b, according to the names from Table 2).

It is interesting to note the increasing overlapping of peaks 2 (SCF) and 2b (substrate) with the increasing thickness of the SCF. The distance between both peaks was 26 °C for 15 µm and just 14 °C for 78 µm. This can be attributed to the shift to higher temperatures of peak 2 originating from the alpha irradiation of the SCF layer, caused by the slower heating up of the surface of the detector with a greater thickness.

**Figure 13:**
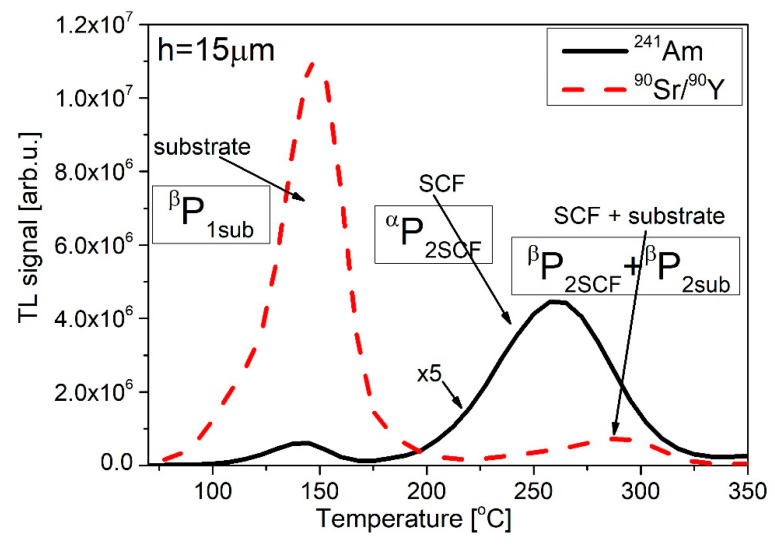
Schematic presentation of the relation between peaks, different parts of detectors, and type of ionizing radiation for LuAG:Ce SCF/YAG SC (sample #1).

In Figure 13, schematic relation between peaks, different parts of detectors, and type of ionizing radiation for LuAG:Ce SCF/YAG SC are presented.

For the derivation of the formulas describing the alpha and beta components of the signal, several assumptions have to be made (see Figure 12 for an explanation of the denotations of peaks):

Peak 1 is related only to the substrate (P_1_ = P_1sub_);

Peak 2 is related to both the single-crystalline film and substrate (P_2_ = P_2sub_ + P_2SCF_);

The signal from the single-crystalline film in the quasi-mixed field is related to both ^241^Am and ^90^Sr/^90^Y radiations.

For simplification purposes, we neglected the contributions of ^241^Am photons.

Thus,

^mix^P_1_ = ^mix^P_1sub_ = ^β^P_1sub_ (3)

^mix^P_2_ = ^β^P_2sub_ + ^β^P_2SCF_ + ^α^P_2SCF_ (4)

where the value of ^mix^P_1_, ^mix^P_2_, ^α^P_2SCF_, and (^β^P_2SCF_ + ^β^P_2sub_) are the maxima of the individual peaks (sub—substrate and SCF—thin film).

By analyzing the glow curve following the irradiation only with ^90^Sr/^90^Y beta particles (which may be considered as the calibration of a detector), we can define the coefficient k describing the P_2_/P_1_ peak ratio:

k = ^β^P_2_/^β^P_1_ = (^β^P_2sub_ + ^β^P_2SCF_)/^β^P_1sub_ (5)

From Formula (4), the contribution from alpha particles (from SCF) can be calculated:

^α^P_2SCF_ = ^mix^P_2_ − (^β^P_2sub_ + ^β^P_2SCF_) (6)

From Formula (5), the expression (^β^P_2sub_ + ^β^P_2SCF_) can be calculated and replaced in Formula (4):

^α^P_2SCF_ = ^mix^P_2_ – k × ^β^P_1sub_ (7)

In dosimetry measurements, one is interested in obtaining values of the quantities, which characterize the unknown radiation field, i.e., the dose or the fluence. In order to retrieve these values from the TL signal of composite detectors, they first need to be calibrated with the relevant radiation.

The calibration factors, s (for beta radiation field) and t (for alpha particles), were determined, following irradiations with the known amounts of radiation:

s = ^β^P_1sub_/D_β_ (8)

t = ^α^P_2SCF_/Φ_α_ (9)

where D_β_—the dose of beta rays and Φ_α_—the fluence of alpha particles.

The unknown beta dose and alpha fluence of the quasi-mixed field can then be calculated using the calibration factors determined in advance.

Because we assumed that peak 1 originates entirely from the dose deposited by beta particles in the substrate, we may write:

D_β_ = ^β^P_1sub_/s = ^mix^P_1_/s (10)

and

Φ_α_ = ^α^P_2SCF_/t = (^mix^P_2_ – k × ^mix^P_1_)/t (11)

Table 4 presents the results of calculations performed according to Formulas (10) and (11). The values obtained for beta doses are, in all cases, in very good agreement with the true values (within 3%). In the case of the alpha-particle fluence, a good agreement (6% difference) was achieved for the composite detector with the thinnest SCF layer of 15 µm. For the thicker SCF layers, the measured values overestimated the true one (up to 53%). The best performance of the composite detector with the thinnest SCF layer was not a surprise, considering that alpha radiation stopped within the first few micrometers of a crystal, and any signal accumulating in the remainder of the detector was just a disturbing factor.

## 4. Conclusions

This work aimed to demonstrate the possibility of creating composite thermoluminescent detectors based on the single-crystalline films (SCFs) and single crystals (SCs) of garnet compounds. The idea of this type of detector is to enable the simultaneous registering of different components in mixed radiation fluxes, namely the fluxes containing α and β particles and X-rays or γ-quanta.

The TL properties of LuAG: Ce SCF/YAG SC detectors were examined under excitation with α particles, β particles, and soft X-rays. TL signals coming from LuAG: Ce SCFs and YAG substrates were found to exhibit significant differences with regard to both spectral and temperature characteristics. These differences might be exploited for the separation of weakly and deeply penetrating radiation in the composite detector.

In practical dosimetry measurements, the spectrally resolved TL data are normally not available. However, analysis restricted only to TL glow curves proved to sufficiently separate the above-mentioned types of radiation. For the mixed alpha/beta fluxes, the evaluation accuracy of doses of both components reached a few percent (for the best 15 µm-thick SCFs). For this reason, the developed composite thermoluminescent detectors based on garnet compounds seem promising new techniques for dosimetry measurements of the mixed radiation fields. Their possible fields of application comprise, e.g., nuclear medicine, where the alpha and beta particles emitters are used, or the diagnostics of other radioisotopic sources.

The discussed encouraging results were obtained for the specific combination of materials of the two layers of the composite detectors. Nevertheless, this work should be considered as a general proof of concept of the whole idea of using this kind of LPE-grown composite detectors for distinguishing the components of radiation fields. The studied LuAG: Ce/YAG combination is certainly not the only possibility to be used for this purpose. It seems reasonable to assume that through investigating other compounds and optimizing their doping and growth conditions, the detectors providing an even better separation between radiation components and characterized by a higher sensitivity might be developed. Such work, comprising various garnet crystals, as well as some other compounds, such as perovskites and silicates, is underway.

## Figures and Tables

**Table 1:** Characteristics of the investigated SCF samples.

Sample Number	Material (SCF/SC)	Density of SCF [g/cm^3^]	Thickness of SCF [µm]	Range of Alpha Particles Energy 3.5 MeV in LuAG [µm]	10 keV Photon Absorption in LuAG [%]	60 keV Photon Absorption in LuAG [%]
#1	LuAG: Ce/YAG	6.71	15	7.80	47	1.5
#2			37		79	3.8
#3			78		96	7.8

**Table 2:** Summary of the positions of TL peak maxima on the temperature and wavelength scales. The data are based on the spectrally resolved measurements after ^90^Sr/^90^Y (SC) and 15 kV X-ray (SCF) irradiations.

Peak Number and Origin of the TL Signal: SCF or SC	Temperature [°C]				Wavelength [nm]			
	substrate	#1 h = 15 µm	#2 h = 37 µm	#3 h = 78 µm	substrate	#1 h = 15 µm	#2 h = 37 µm	#3 h = 78 µm
1 (SC)	144	138	144	140	700	702	708	708
2a (SC)	240	245	240	-	800	803	804	-
2 (SCF)	-	268	290	284	-	533	538	542
2b (SC)	300	300	295	-	700	711	713	-

**Table 3:** Peak parameter estimations using TL glow deconvolution.

	P1	P2	P3	P4	P5
^90^Sr/^90^Y, h = 78 µm					
T_max_ [°C]	105	148	170	-	282
E [eV]	1.24	1.09	0.88	-	0.94
A	86,283	1,161,109	134,947	-	178,764
^241^Am, h = 78 µm					
T_max_ [°C]	87	143	158	267	296
E [eV]	1.24	1.09	1.03	1.2	1.11
A	1200	29,723	2838	156,770	53,573

**Table 4:** Calculations of the contributions of alpha particles and beta radiation to the quasi-mixed field. Reference values: Φ = 1.58 × 10^11^ [cm^−2^] and D_β_ = 120 [Gy].

Sample Number	Thickness of SCF [µm]	^calc^Φ_α_ [cm^−2^]	^calc^D_β_ [Gy]	^calc^Φ_α_/^ref^Φ_α_	^calc^D_β_/^ref^D_β_
#1	15	1.91 × 10^11^	119	1.06	0.99
#2	37	1.97 × 10^11^	116	1.24	0.97
#3	78	2.41 × 10^11^	119	1.53	0.99

## Data Availability

Not applicable.
